# Reduction, alignment and visualisation of large diverse sequence families

**DOI:** 10.1186/s12859-016-1059-9

**Published:** 2016-08-02

**Authors:** William R. Taylor

**Affiliations:** Francsis Crick Institute, 1 Midland Rd., London, NW1 1AT UK

**Keywords:** Sequence clustering, Multiple sequence alignment

## Abstract

**Background:**

Current volumes of sequence data can lead to large numbers of hits identified on a search, typically in the range of 10s to 100s of thousands. It is often quite difficult to tell from these raw results whether the search has been a success or has picked-up sequences with little or no relationship to the query. The best approach to this problem is to cluster and align the resulting families, however, existing methods concentrate on fast clustering and either do not align the sequences or only perform a limited alignment.

**Results:**

A method (MULSEL) is presented that combines fast peptide-based pre-sorting with a following cascade of mini-alignments, each of which are generated with a robust profile/profile method. From these mini-alignments, a representative sequence is selected, based on a variety of intrinsic and user-specified criteria that are combined to produce the sequence collection for the next cycle of alignment. For moderate sized sequence collections (10s of thousands) the method executes on a laptop computer within seconds or minutes.

**Conclusions:**

MULSEL bridges a gap between fast clustering methods and slower multiple sequence alignment methods and provides a seamless transition from one to the other. Furthermore, it presents the resulting reduced family in a graphical manner that makes it clear if family members have been misaligned or if there are sequences present that appear inconsistent.

**Electronic supplementary material:**

The online version of this article (doi:10.1186/s12859-016-1059-9) contains supplementary material, which is available to authorized users.

## Background

### Introduction

Many sequence analysis methods, such as correlated mutation analysis [1] rely on having large multiple sequence alignments. However, others benefit from having a more balanced representation, not only to avoid bias towards well populated subfamilies but also for ease of visualisation. (see Note 1) As the volumes of sequence data continue to grow at an ever increasing rate, the problem of reducing a large diverse family to a representative selection is becoming an important requirement.

The selection of a representative subset of sequences is most simply achieved by calculating the pairwise similarity of the sequences and setting a cutoff, say, 80 % identity, and selecting one member of the pair as a representative. A more complex algorithm has been proposed for protein structure based on finding representatives that are equally spaced, given some distance metric. This worked by applying cycles of culling to remove the proteins with most neighbours (within a given cutoff) until only isolated representatives remain [2].

With large numbers of sequences, two problems arise. The first is purely practical: if the sequences are not aligned the pairwise calculation of similarity can be computationally very expensive and even if the sequences are aligned, with very large families (which may exceed 100,000 members) just the evaluation of the similarity can be a burden. The second difficulty arises less from computational limitations and more from the problem of which criteria should be used to select a representative sequence from a pair or subfamily.

The practical step of comparing many sequences has been approached through the use of peptide (or oligonucleotide) counting statistics which have been used as a heuristic for sequence similarity for many years [3, 4] and have found applications not only to protein sequences [5–9] but also to the large volumes of next-generation-sequencing data [10–14]. Despite these many varied and often very fast algorithms, peptide based sorting can only provide a rough estimate of the similarity between sequences and, if computationally possible, it is always better to use a full alignment method or preferably, a profile/profile comparison method for multiple sequences.

There are many criteria that can be considered in selecting a representative sequence. Perhaps most important is to avoid errors such as premature truncation or missing segments. Although not strictly an error, many databank search programs are prone to lose positions at the termini of sequences where the degree of similarity tends to be weak and this problem of "terminus shrinkage" can be undesirable especially where it can lead to loss of a long-range (sequence-wise) interactions that can be important in structure prediction [15].

Equally important, but not so easily quantified, is the benefit of selecting sequences that have the best annotation and most importantly, for homology modelling, have a known structure. The existence of a known structure, depending on the sequence database, can be trivially identified as having an identifier associated the the Protein Structure Database (PDB). Without taking the time consuming step of making links to external databases such as the Gene Ontology database, the information included in the sequence title is generally all that is available. From this, however, useful key-words can be recognised such as: "hypothetical" or "mutant" that can be scored and used to down-weight the selection probability for the sequence.

### Outline

In this work, I present a method, based on an existing multiple sequence alignment program [16], that incorporates these features and uses a fast peptide (or nucleotide) based sorting algorithm to generate an ordered list of sequences before any pairwise comparison is performed, similar to the way in which a peptide based method was used previously to ‘feed’ iterated multiple sequence alignment [5, 17]. As this is used only in the current work as a preliminary step which is followed by full sequence alignment, the method adopted below therefore used the computationally least demanding implementation that still retained sufficient fidelity. The method has the capacity to accept the results generated by typical search methods, such as BLAST [18] and JackHmmer [19], even when the number of "hits" approaches 100 thousand.

The aim of MULSEL is to produce a small alignment of representative sequences that can be annotated with derived secondary information (such as secondary structure prediction) to inform on the consistency and validity of the alignment. It does this through a series of hierarchical reductions (or condensations) into mini-alignments and at each stage a representative sequence is selected from each mini-alignment to be passed to the next stage of the condensation. This selection process is based on some generic properties of the sequence (eg, length) but is augmented by user specified criteria, such as quality of annotation (say, indicating a known structure) or species or any quality the user thinks important to their study, including the ability to add a bias to include sequences that have been marked by the user, irrespective of any annotation, as being of special interest.

In the following sections, the robustness of this approach will be tested and compared to pairwise comparison based methods (BlastClust and CD-hit). To do this requires a set of sequences that contain sub-families that include members reaching to the limits at which sequences similarity can be detected but with the additional constraint that there should be no false connections made between members of different families. Test data of this nature could be constructed by combining sequences from two biologically distinct families. However, it is difficult to find challenging examples with pairs of families that lie at the limits of separation as it is then often unclear whether the two families are distinct (e.g., haemoglobins and myoglobins).

This difficulty was overcome in two ways by generating artificial sequence families and pseudo-artificial families. The first approach was to construct sequences that have an over-representation of one particular amino acid type and the second is to use real biological sequence families but combine each with a family of "anti-sequences" which are the same sequences but in reversed order. (Meaning that the actual sequence of amino acids is reversed in every protein, not the order of the sequences in the input file). In the first approach, clearly, 20 distinct sequences will remain in the reduced selection whereas in the second, just a sequence/anti-sequence pair will remain.

In principle, the hierarchic condensation through mini-alignments can begin with sequences in any order but to reduce the amount of computation required using a full pairwise dynamic programming alignment algorithm [20], it is much better if the starting sequence order has similar sequences in neighbouring positions. This means that the number of sequences taken to form each mini-alignment can be kept small. However, the order does not have to be exactly optimal because, even if a pair of similar sequences is missed at an early stage, they should be brought together at some point in the condensation and one will be selected in preference to the other. (Even if this does not occur, having a pair of similar sequences remaining in the final alignment is not a problem, as long as there are not too many of them).

(Note: Throughout the text, the term “family” will be used to refer to a group of sequences that can be aligned together at a given cutoff level of a similarity score but that cannot be aligned with another family at the same cutoff level. The term “subfamily” will then be a group of sequences within a family that obey the same rules but with a stricter cutoff. As this is just an operational definition it contains no biological or evolutionary implications and will vary with each comparison method and score matrix and gap-penalty).

## Results

### Test data

The operation of the method was tested using the artificial families generated by random mutation of 20 seed sequences, with each starting as a homopolymer of all twenty amino acid types. The data was designed so that all sequences that are enriched in the same amino acid can be aligned with each other but not with a sequence that has been enriched with a different amino acid type. (See “Implementation” section for details). This means that when sequences from the same acid-rich family are brought together in the same mini-alignment, then one will be selected and repeating this process will result in just one sequence from each family remaining, giving a final collection of 20 distinct sequences. Any failure in the sorting or condensation stages will result in a final collection of more than 20.

An equivalent test was then carried out using real (native) sequences combined with their reversed “anti”-sequence dopplegangers. If successful, this test should result in just two distinct sequences remaining.

#### Artificial sequence families

A minimal protocol was tested consisting of one pass of the peptide presort (using a tetra-peptide length) followed by increasing numbers of mini-alignment/selection cycles, each to a limit of 50 % residue identity. (To be exact, the cutoff of 50 is the score obtained for a profile/profile comparison using an identity matrix. For the small numbers of related sequences in the mini-alignments this will fall only slightly below a simple pairwise score). Each alignment cycle used a *span* parameter setting of 50, meaning that sequences separated by more than 50 in the input order are not compared. (See the “Program Implementation” section* in the Additional file 1 for details). Thus, if the presort step has not clustered related pairs within this range, they will not be aligned until there is 50 or fewer mini-alignments between them.

On the 10 K sequence test data set (see Implementation), the results show that the bulk of the time is spent on the alignment stages with only 30 seconds taken by the initial peptide pre-sort. After four rounds of alignment no further gain is made, at which point, the 10 K sequences have been reduced to 172 but these are grouped into 151 families and subfamilies which is well in excess of the target 20. Clearly, more emphasis needs to be shifted towards the faster peptide pre-sort rather than the alignment stage (Table 1*a*).

**Table 1:** Segregation success and run-times for different clustering strategies are tabulated for: *a*) a single peptide pre-sort with increasing numbers of alignment cycles, *b*) staged alignment cycles from 90 to 50 % identity each with a peptide presort (using 1 pass), *c*) as for (*b*) but with multiple pre-sort cycles indicated in parentheses)

*a*	Single pre-sort		
Alignment	Time	Sequences	Remaining
stages (to 50 %)	sec.	selected	subfamilies
1	58.8	1658	503
2	91.4	355	302
3	226.1	196	171
4	462.6	175	154
5	727.1	172	151
*b*	Staged pre-sort		
Alignment	Time	Sequences	Remaining
stages (3 to X%)	sec.	selected	subfamilies
90 (1)	36.30	1597	947
80 (1)	1.33	563	314
70 (1)	1.00	165	31
60 (1)	0.53	104	24
50 (1)	0.47	71	21
*c*	Staged (multi-pass) pre-sort		
Alignment	Time	Sequences	Remaining
stages (3 to X%)	sec.	selected	subfamilies
90 (8)	10.62	3641	598
80 (4)	4.26	1034	93
70 (2)	2.31	285	40
60 (1)	1.10	98	20
50 (1)	0.42	62	21

The data columns indicate the elapsed time in seconds (real time reported by the Linux time utility), the number of the 10,000 starting sequences remaining after each stage and the number of families or subfamilies (defined by sequence adjacency

Additional fast sorting stages were introduced by alternating these with sequence alignment stages and with each alignment stage, the threshold above which pairs were clustered was gradually reduced from 90 to 50 % in stages of 10 %. As the initial alignment stages are only considering closely related sequences, their alignment parameters can be constrained to allow inserts of only several positions and the range of adjacent sequences (*span*) be reduced, as any failure to find a match at the early stages will be corrected at a later stage. The times taken for each stage are now greatly reduced, with the peptide presort now becoming the limiting factor in the early stages. Importantly, the number of final sequences has now almost reached the expected 20, and after a final multiple sequence alignment, only 20 family representatives remain (Table 1*b*).

To redistribute the initial load carried by the first peptide presort, this was partitioned using the blocked-diagonal methods described in the “Implementation” section using 8 blocks at the 90 % stage, 4 at the 80 % and 2 at the 70 %, with the following 60 and 50 % stages evaluating all pairs. In the first three passes, the load balance shifted from 36:1:1 to 10:4:2 (over cycles 1:2:3), with the overall time halved from 39.6 to 18.7 seconds (Table 1*c*).

This protocol was tested on the larger test sequence sets of 30 and 90K sequences (Table 2, *a* and *b*) in which a similar rate of condensation was observed with both runs reducing the number of subfamilies to 22. The reason why the target number of 20 families failed to be reached was investigated and found, in both cases to be due to two isolated outliers in sequence composition, one of poly-A and the other poly-E, that failed to align with their expected A-rich and E-rich families. This was not due to a failure to be considered but because the larger profiles for each family were now so diverse that the two homopolymers failed to score more than the cutoff.

**Table 2:** Segregation success and run-times for large sequence collections are tabulated as in Table 1

*a*	30,000 sequences			
Alignment	Time	Sequences	Remaining	cd-hit
stages (3 to X%)	sec.	selected	subfamilies	clusters
90 (8)	126.2	10892	1085	1152
80 (4)	32.2	3352	250	588
70 (2)	10.6	929	67	377
60 (1)	4.5	302	22	253
50 (1)	1.8	203	22	173
*b*	90,000 sequences			
Alignment	Time	Sequences	Remaining	cd-hit
stages (3 to X%)	sec.	selected	subfamilies	clusters
90 (8)	1695.8	33734	3234	3562
80 (4)	323.4	10740	794	1861
70 (2)	70.3	2899	182	1154
60 (1)	19.8	947	49	771
50 (1)	6.1	634	22	525

The final pass by MULTAL to align the remaining sequences correctly generated the 20 distinct families for each sequence collection. The reason why MULSEL left two singleton unclustered sequence “subfamilies” is explained in the text. The results of the fast clustering program cd-hit are included for comparison but it should be noted that these values are not directly comparable as cd-hit is a single pass method whereas MULSEL is progressive. The values quoted are the smallest number of clusters reported for all combinations of parameters that produced a result with the cutoff (-c) from 0.4 to 0.9 and word lengths (-n) from 5 down to 2

The times to reduce these large sequence collections (10, 30, 90K) to a manageable size, of 0.3, 2.9 and 35.2 minutes (elapsed time on a laptop) are well within the range that is acceptable for routine sequence analysis. Most of this time is taken by the sequence alignment stages following the peptide pre-sort and, despite the efficiency of the peptide sorting stage, the times still have a quadratic dependency on the number of sequences (*N*^2^/4, seconds for *N* thousand sequences) so sequence volumes over 90K would be best calculated initially in parts as suggested above or by using a fast program like cd-hit as a pre-filter. This method follows a similar short peptide based sorting strategy but invests less time in the alignment stage (using only pairwise alignment) and as such does not find such remote sequence similarities, reducing the 30K and 90K collections to 173 and 525 clusters, respectively, rather than the target 20 (Table 2).

An alternative approach to clustering large numbers of sequences takes advantage of the fast search and alignment algorithm in the blast program combined with single-linkage clustering (blastclust v2.2.26 (ftp://ftp.ncbi.nlm.nih.gov/blast/executables/LATEST). However, blastclust is not intended to reduce the sequence selection so direct comparison is not totally valid. Nevertheless on the same test data, the times required by blastclust (on the same laptop, with default parameters) were: 11.9, 110.3 and 876.2 minutes. The latter time (over 14 hours) would not be ideal if repeated runs were required. The number of clusters returned by blastclust were in excess of 1000, somewhat above the expected 20. Although the parameters had not been adjusted for the degree of sequence divergence in the test data, even if this were improved, the heavy computation time makes the method less attractive.

#### Peptide length and alphabet reduction

Using the smallest (10 K) test data set, the effects of changing the peptide length used in sorting and the reduction in the number of distinguished amino acids (referred to as ‘softening’ above) were investigated. The results in Table 3 for various combinations of these parameters show that a peptide length of 5 or 6 is optimal but that reducing the alphabet is not advantageous. However, as the test sequences were not evolved under a model that reflected amino acid similarity, this is not unexpected. The computation times were relatively unaffected by the parameter choice (Table 3).

**Table 3:** Segregation success and run-times (in parentheses) for different combinations of peptide lengths (used to calculate sequence similarity) and the degree of ‘softening’ used to reduce the amino acid alphabet

Soft	Peptide length				
	3	4	5	6	7
0	21 (10.26)	21 (10.64)	20 (10.96)	20 (11.12)	20 (10.81)
	23 (124.5)	22 (125.0)	21 (121.1)	20 (115.5)	31 (111.2)
1	25 (10.33)	24 (10.70)	20 (10.96)	24 (11.15)	24 (11.04)
	34 (127.7)	26 (125.7)	33 (124.1)	35 (117.6)	79 (125.1)
2	24 (10.45)	22 (10.90)	24 (11.12)	24 (11.28)	28 (11.16)
	43 (128.7)	36 (128.0)	43 (126.1)	50 (121.2)	71 (130.0)
3	33 (10.44)	29 (10.84)	29 (11.23)	30 (11.60)	28 (11.35)
	60 (129.8)	58 (130.9)	69 (127.4)	59 (122.5)	75 (132.0)
4	31 (10.31)	31 (10.92)	32 (11.12)	35 (11.62)	36 (11.42)
	69 (132.3)	75 (132.0)	71 (130.0)	79 (125.1)	69 (132.3)

Two sets of values are reported for each combination corresponding to starting sets of 10 and 30K sequences. The values are the number of final families and the time in seconds taken for the first peptide based pre-sort

Two parameters remain to be tested which are: the cutoff on the minimum score below which a pair are not stored and the number of top matches retained per sequence. The nature of the test data is not suited to testing the first as homopolymers can only ever score 1 and tests on the second indicated that saving just the top hit may be sufficient but that the time saved is not significant. These parameters were also tested on real sequence data (below) but had little effect on the quality or execution time of the algorithm (data not shown). As such, they were kept at their ‘default’ values of 3 top hits held per protein with any score under 10 discarded.

#### Pseudo-random sequence families

Tests were carried out using the data generated from sets of native sequences combined with their corresponding reversed “anti-sequence” partner, or doppleganger (a device originally used to test motif matching [21, 22]). Although this may seem to be a trivial test, the sequences and their reversed partners are unexpectedly similar as they have the same length distribution and sequence composition, which combined with directionally symmetric secondary structure elements (and super-secondary structures), leads to an intrinsically high, but unspecific, background similarity.

Using the PFAM family PF00072 [23, 24], which is the chemotaxis-Y protein family (described in more detail below), sets of 600 to 30,000 sequences were extracted (counting reversed dopplegangers). The equivalent result to that performed above is to reduce each set to two members with one native and one reversed representative remaining. These sets were processed by MULSEL and the number of selected sequences was monitored at the point just before either the native sequences or their dopplegangers were all gone. At this stage, the target pair of sequences remained in four of the five runs with three of each sequence type (true/anti) remaining in the fifth. Neither CD-hit or BlastClust produced any significant degree of clustering on these remotely related data sets, with the best reduction being CD-hit which made 345 clusters from 600 starting sequences.

The massive reduction of sequence numbers by MULSEL to a few representatives, starting from several thousand sequences, is an encouraging test but does not represent how MULSEL would normally be used, which is to halt at around 100 sequences and then perform a full multiple sequence alignment. The previous tests were therefore rerun with a condition to stop when the reduced number of sequences fell below 100 and the sequences aligned. The resulting sequence order was then examined to see how cleanly the true sequences had partitioned from their reversed dopplegangers. In all-bar-one of the five runs, a clean division between true and anti-sequences was obtained. (See Additional file 1 for the complete output with dendrograms). In the other run, just a single pair true sequences had aligned with a pair of anti sequences at an early stage in the multiple sequence alignment. However, in a another run, although the ordered list of sequences was split in half, the dendrogram consisted of three major sub-trees, the middle of which was split between reversed and native sequences (See alignment in Additional file 1).

Whatever the composition of the sequence collection, if they remain distinct from their reversed members, then this is a strong indication that they can be treated as a single family. Where outliers become entangled with their dopplegangers then they should be treated with caution and in the split sub-tree described above, the sub-family of true sequences could either be excluded automatically from the family or aligned and assessed using the visualisation tools described in the “Implementation” section and illustrated in the following sub-section.

### Biological sequence data

#### Globular protein

One of the larger protein sequence families is typified by the small chemotaxis-Y protein (cheY) which has often been adopted for test purposes [25, 26]. A scan over the NCBI non-redundant protein sequence database, with a query sequence taken from the PDB structure entry 3chy, was made using JackHmmer [19] with three iterations at a cutoff E-value level of 0.0001. This found 353,564 similar sequences, which is well over the number that can be processed by MULSEL. However, as mentioned above, the sequences can be pre-processed in batches and recombined when their total has been reduced to the required level. The search results were split into eight batches of 44K sequences and reduced down to the 70 % level, with each batch (run in parallel) taking 10 minutes. (For protein sequences, the score is now evaluated using an amino acid exchange matrix and cannot be simply interpreted as the percentage of matching characters).

**Fig. 1:**
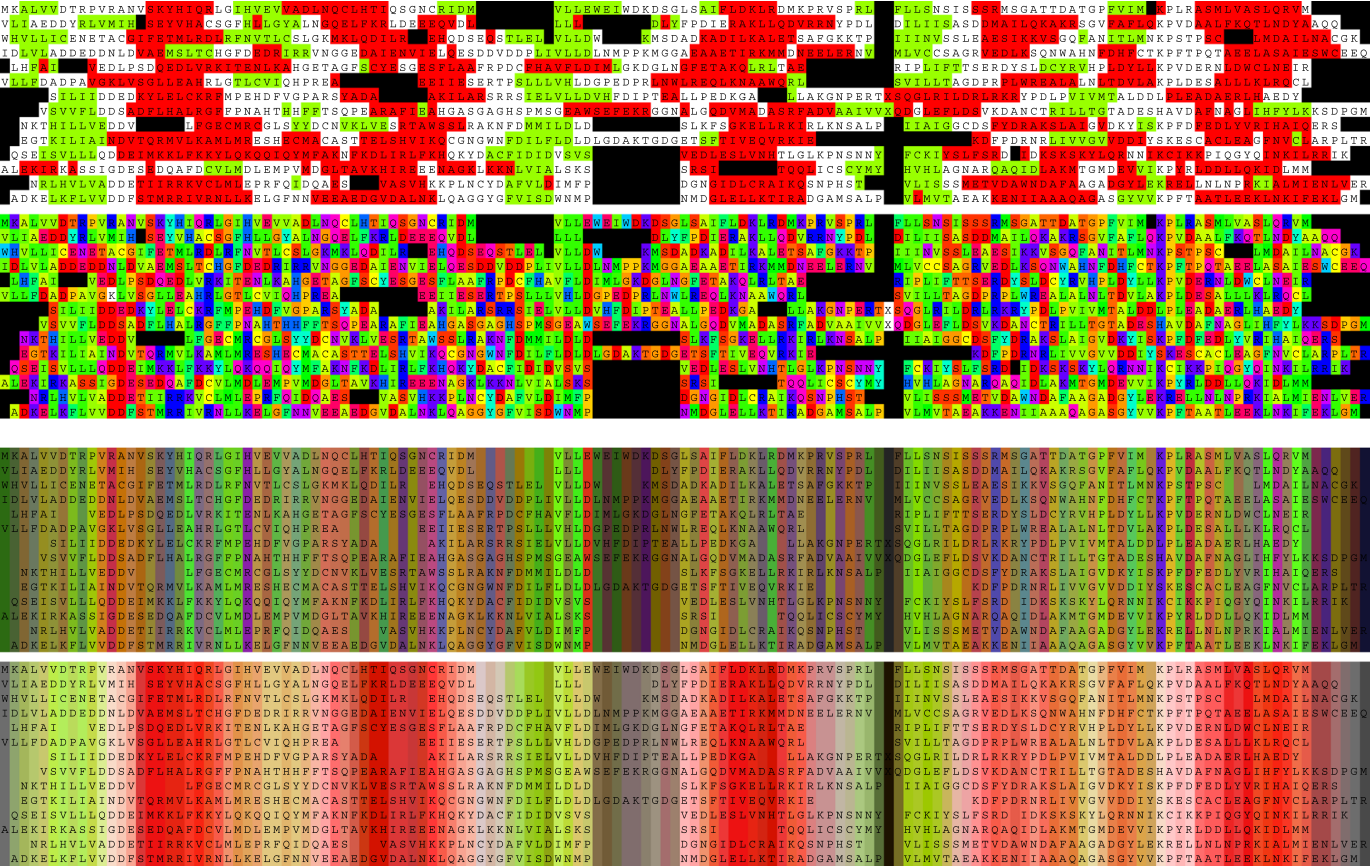
Chemotaxis Y protein family. The 14 sequences remaining after reduction from over 350,000 were aligned and coloured as described above (Sect. 2.5) with the top two panels coloured by individual residue secondary structure state (*red*= *α*, *green* = *β*) and amino acid identity (phobic = *green*, polar = *red* (-ve) to *blue* (+ve), approx.). The lower two panels ‘reflect’ the top two but use average colours to emphasise conservation

The selected sequences were combined, forming a collection of just over 70 K sequences which was reduced again down to a collection of 1665 sequences after a first pass at the 40 % level and cycles at this level were repeated until the number of sequences was further reduced. Normally, a family of up to 100 sequences would be retained so as to preserve a good spread of variation but to reduce the size of the printed alignment (while keeping a font-size that remains legible), the family was reduced until it reached 14 at which point they were aligned (Fig. 1). A feature of the output that is not captured in these coloured plots is the number of sequences represented by each selected sequence, however, this is written into its annotation as the number between “[+...+]” (as seen in the Additional file 1). When assessing the quality of the alignment, any deviant sequence representing few or no others should be considered as a candidate for removal before another representing thousands.

It can be seen clearly from Fig. 1 (top panel) that the family, correctly, appears to be an alternating *β*/ *α*-type protein. However, guided by the secondary structure predictions, some segments could clearly be realigned to increase the consistency of these predictions — such as the first *β*-strand. Many methods have been devised that use such derived information to modify the alignment by local changes in gap-penalty and amino acid substitution [27–32]. However, none were applied in this example.

#### Transmembrane protein

A similar protocol was followed with a rhodopsin sequence as a search query. This protein is a member of the large G-protein coupled receptor family and is commonly used as a test for prediction and modelling [33]. Using the Jackhmmer program with the sequence of known structure (PDB code: 1GZM) as a query (omitting the first 20 residues that are disordered in the structure). A search extracted 183,872 sequences with a cutoff value of 0.00001 after 4 iterations. Rather than process the complete collection in batches, the JackHmmer alignment was reduced to delete all positions that were gapped in the query sequence and from the resulting alignment, all sequences with over 25 % gaps against the query were then removed leaving 27,024 sequences, which is easily within the scope for processing with MULSEL.

**Fig. 2:**
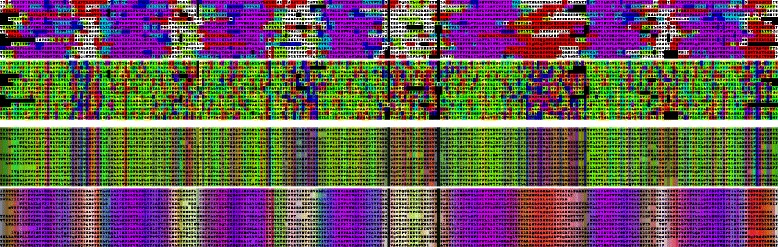
Rhodopsin family. Over 180,000 rhodopsin sequences reduced to 14 coloured as Fig. 1 but with an added *blue component* to indicate predicted TM segments. As these typically overlay *red α-helix* predictions, the resulting *purple hue* can be taken to identify the seven TM-segments which are even clearer in the averaged colours (*lower panels*)

The final alignment of, coincidentally also, 14 sequences was coloured using not only their predicted secondary structure state but also their predicted transmembrane segments (see “Implementation” section). The seven transmembrane segments with their corresponding hydrophobic amino acids (coloured green) can clearly be identified (Fig. 2). Unlike the previous example, a version of MULTAL [33] was used that increases the gap-penalty in TM-segments and also applies a specific amino acid exchange matrix when aligning TM-segments [34] while retaining an exchange matrix more suited to globular proteins in the loop regions [35].

#### RNA

**Fig. 3:**
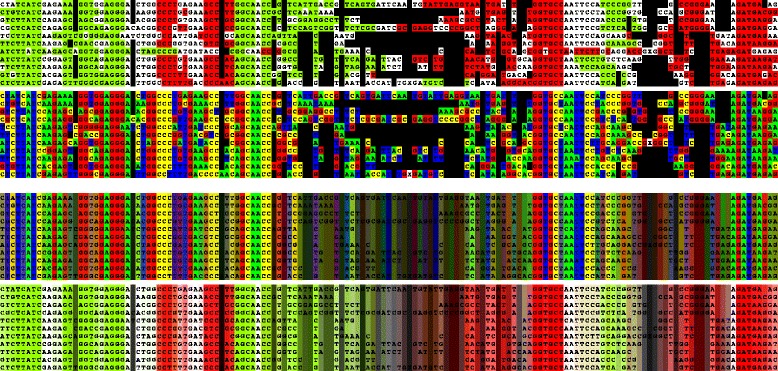
SAM-III riboswitch RNA family. The family reduced from over 400 sequences is depicted as for the protein examples above but with the exception that the predicted stem-loops are coloured *green* when descending into a hairpin and red when ascending. The nucleotide colours are: *red*, *green*, *yellow*, *blue* for G,A,C,T respectively. (N.b., Uracil is recoded as ‘T’ to retain the 20-letter amino acid alphabet)

As a example of the approach applied to an RNA family, the sequence of the SAM-III riboswitch was taken. However, as the MULTAL/MULSEL parameters are not optimal for nucleic acid sequences, the aligned family was taken directly from the Rfam database (family RF00162) [36]. With just over 400 sequences, the family presents little of a computational challenge and was reduced in a matter of seconds to a representative selection of 11 sequences. To avoid realignment by MULTALMULSEL, the gap characters in this alignment were replaced by ‘X’ characters and a high gap-penalty imposed with the default identity scoring matrix. (The ‘X’s were then returned to gaps before visualisation). By colouring strands differently depending on whether they are entering or exiting a stem-loop, the overall clover-leaf secondary structure of this molecule can be identified easily (Fig. 3).

## Implementation

### Peptide-based presort

For a given peptide length (typically, 3 or 4), a binary tree is created in which each overlapping peptide in a sequence (encoded by a unique numeric identifier) is allocated to a leaf-node in a binary tree. Unlike algorithms that allocate space for every possible peptide, a binary tree only allocates space for observed peptides and is therefore not limited by the length of the peptide.

For simplicity, a fragment of sequence will be referred to as a “peptide”. However, the algorithms described below treat all characters equally using the alphabet of single-letter amino acid codes. This means that the RNA base, uracil (U) should be encoded as ‘T’. The smaller nucleic acid alphabet will generally require a longer “peptide” length to behave in a similar way to proteins.

**Fig. 4:**
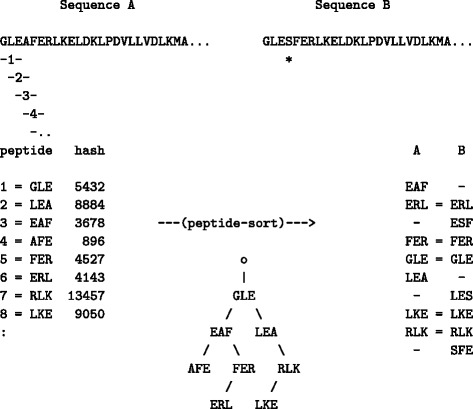
Peptide-based sequence score. Two protein sequences, *A* and *B*, are compared (which differ only in the A →S substitution marked by an asterisk). Tri-peptides are extracted and sorted using a binary tree (in NlogN time). For simplicity, only the first 10 positions in sequence A are shown and the peptides are represented using the one-letter amino acid code, rather than their hash value (big numbers). The tree is parsed in a depth-first, left-right order (starting at the root node “o”) with the node value (peptide) being written just once when first leaving upwards (u) or down-right (r). The ordered peptide list is: AFEu, EAFr, ERLu, FERu, GLEr, LEAr, LKEu, RLKu, with the lower-case suffix indicating the condition on which the peptide was written. The two lists of sorted peptides for sequences A and B can be scanned for common entries in linear time (*right side*). The same code was also used for pairs of scored sequences with the numeric value being the score for the pair. If only the M highest pairs are to be stored for each sequence, then as the tree is loaded, if M higher pairs are encountered (*left moves*) the pair can be skipped and at the end, just the M highest entries extracted. In the peptide example: if M=2, then AEF would not be entered (or ERL)

The sorted list of peptides were then extracted in ranked order and used as a reduced representation of the protein for fast pairwise comparison. This step employs a recursive routine and is equivalent to the quick-sort algorithm, taking in the order of *L* log(*L*) time in the length, L, of the sequence (Fig. 4).

As the peptide lists are ordered, two proteins can be compared without alignment as a simple count of their common peptides which can be obtained with a single pass over the joint lists (in linear time). Ideally, this value should be stored for all pairs to be used later in clustering but for a large number of sequences, *N*, this would have a quadratic time and storage dependency and is best avoided.

Two devices were used to reduce the (*N*^2^−*N*)/2 storage requirement. Firstly, the simple step of applying a cutoff to discard low-scoring pairs was used, and secondly, only the top *M* similarities were held for each protein, giving an *N*×*M* requirement, with *M* typically just 3. The additional computational complexity of resorting each list of hits every time a new pair was evaluated was treated in a similar way to the peptide sorting with the use of a binary tree construct. (See Fig. 4). (See also the source-code routine seqsort.c in the Additional file 1 for further details).

After all pairs of sequences had been compared, the *N*×*M* top scores were extracted from the tree structure into a sorted list (without additional computation). These ordered pairs, beginning with the highest, were then used to grow clusters by a single-linkage cluster algorithm (using the same code as the original MULTAL program [37]) which requires only one pass over the list of ranked pairs.

Lists of sequences extracted from a databank search are seldom in random order, with adjacent sequences commonly having some similarity. To capture this, adjacent sequences in the input list were given a small bonus to their pairwise score that helps to preserve the original sequence order in the absence of any clear indication to re-sort it.

### Local mini-alignments

Given a list of sequences roughly sorted by peptide composition, the elimination of redundant sequences followed the strategy outlined in the original MULTAL multiple sequence alignment program. As with the peptide pre-sort described above, this calculates a pairwise sequence similarity and aligns those joined by single-linkage clustering. However, as the sequence order is now sorted, only relatively adjacent sequences need to be compared and aligned only if they have a similarity above a predefined threshold. This process generates a series of small local multiple sequence alignments of, typically, between 2 and 10 sequences from which a representative sequence can be selected to generate a reduced list of sequences. As outlined in the Introduction, a number of criteria were employed in this selection. (The maximum number of sequences in a mini-alignmennt depends on the number of alignment iterations (*N*) as it is possible for a pair of sequences/profiles to be aligned at each stage, giving a maximum of 2^*N*^).

If the input includes a sequence of special interest, such as a sequence for which a model or prediction is to be made, this can be identified by including the string “SEED” in its code. (That is this is the string following the “>” character at the start of a sequence entry). The sequence selected from the mini-alignment is then biased to keep a close match to the seed-sequence length. Otherwise, the selection is biased towards a sequence close to the average length of those in the mini-alignment. This was implemented as penalty score, *p*, as *p*= log(*d*^2^+1), where *d* is the deviation from the target length. The penalty has the value 5 at a deviation of 12 residues rising to 9 by 100 residues. When there is a specific seed sequence, this score was modified to give half the penalty value to sequences that are longer than the seed. This bias directs the sequence selection away from fragmentary sequences and helps reduce the “terminal shrinkage” problem mentioned in the introduction.

Additional biases were extracted from both the sequence identification code and other information contained in the annotation. A strong bias (-60) was added for selection of sequences with a PDB code and a lesser bias (-20) for sequences from UNIPROT. A strong bias was added against sequences that had the words: “mutant” (+40) or “fragment” (+50) in their titles and smaller penalties for the words “probable” (+1), “precursor” (+2), “uncharacterised” (+5) and “hypothetical” (+5). If there is an identified seed sequence it is, of course, highly desirable to retain it in the selection and a large penalty (-100) was assigned. Other sequences that may have constituted a seed alignment can be identified by the inclusion of the (lower-case) “seed” key-word in their code and these were also biased for retention (-50).

The units of these penalties are the number of common peptides (unique N-tuples) counted between two sequences. This will be slightly length dependent, for example two sequences of length 3 have a score of 1 and as the sequences become longer, the number of common peptides encountered by chance will increase but because of recurring peptides, not as fast as say, a full alignment score. However, the biases are all imposed within the context of a mini-alignment in which the sequences are all close in length.

After the summation of all the penalties, the sequence with the lowest (best) score is written to the new sequence collection. The whole process is then repeated on this reduced collection, either with or without a reduction in the cutoff threshold that determines the size of the mini-alignments.

### Artificial test data

The starting points for the generation of test data were twenty sequences consisting of just a single amino acid type. Each sequence was then successively substituted with all 26 letters and any non amino acid 1-letter codes (B,J,O,U,X,Z) were replaced by the gap character (‘-’). When the mutated sequence had less than 1/3 of the starting amino acid (33 % is close to the limit that can be detected by sequence similarity), then substitutions were made with the original type until the composition was restored to over 1/3. An example of the sequence test data and a family alignment is shown in the Additional file 1: Figures S1-S3.

Each sequence was assigned a random number which was used to shuffle their order. Collections of 10, 30 and 90 thousand test sequences were generated and their gap-characters removed. Each sequence collection was then passed to MULSEL and a variety of parameter options tested with both the quality of the clustering and the computation time being monitored. (For all tests, the time reported is the real time taken for the process running on a single Intel processor on a moderate laptop as reported by the Linux time() utility).

To assess the speed and quality of the method, the test data described above has the advantage that it contains twenty separate families each enriched with a character in the single-letter amino acid alphabet. As these were limited to no less than 1/3 of their starting identity, they should all align within a family but not between families. If MULSEL has been successful in uniting all members of a family, then at the end, a count of sub-families will equal twenty. The rate of clustering can also be followed by performing this count at intermediate stages.

### Visualisations

Having reduced a large sequence collection to a small multiple sequence alignment, it is important to gain a clear overview of the quality of the alignment and including derived features can help in this assessment. Derived features for different types of sequences are described below while the overall quality of the alignment is aided by colouring amino acids as specified by a physico-chemical based colouring scheme [22] and for nucleotides as specified by Jalview [38]. As previously, these colours are averaged across the alignment in a separate panel, emphasizing conserved positions as bands of saturated colour.

#### Globular proteins

A good indication of a protein’s structure and a guide to the quality of the alignment can be gained from their predicted secondary structure. For this the psipred program was used [39] but rather than scanning each sequence over the full sequence databank (using psiblast [40]), each sequence was scanned only over the original sequence collection, or for even greater speed, only those selected for the final alignment. Each different query returns a slightly different alignment (depending on their degree of divergence from the family) which gives an indication of the degree of variation. As with the amino acid colourings, these predictions were averaged in a separate panel.

#### Transmembrane proteins

For transmembrane (TM) proteins, psipred predictions were made as described above, but in addition, TM segments were predicted using the memsat program [41]. Although later versions of this program have been developed [42], and many alternatives exist [43], the original method uses just a single sequence and so gives an indication of the degree of prediction variation across the family. This can be used as a basis to decide whether the full collection of aligned sequences should be used for a consensus prediction using a more recent method that uses a multiple sequence alignment.

#### RNA

RNA secondary structures were predicted using the RNAfold method from the Vienna Package [44]. Although there is essentially only one type of RNA secondary structure in which the chain forms a base-paired hairpin (stem-loop), some added information was included by using different colours for the descending and ascending strands. This is often sufficient to give an impression of the overall 2D structure. As with the above methods, if the predictions appear consistent, the alignment can be used with a method in the package that calculates a consensus prediction.

## Discussion

Many bioinformatics based research projects either begin or include a search of the sequence databanks with one or more query sequences of interest. With the huge size of the current sequence collections, the number of sequences of potential similarity identified on such searches can be enormous, typically in the range of 10s to 100s of thousands of sequences, depending on the query and the search parameters.

Faced with such a volume of data it is often quite difficult to tell from the raw data whether the results make sense or whether, through profile drift on iterated searches, they may include sequences with little or no relationship to the query. The best approach to this problem is to cluster and align the resulting families and some methods that perform this task have been discussed above. However, existing methods concentrate on fast clustering and either do not align the sequences or only perform a limited alignment, leaving a still considerable task to align and combine the clusters to represent the full range of family diversity.

### Summary

In this work a method was presented that combines fast peptide-based pre-sorting with a following cascade of mini-alignments, each of which are generated with a robust profile/profile method. From these mini-alignments, a representative sequence is selected, based on a variety of intrinsic and user-specified criteria that are combined to produce the sequence collection for the next cycle of alignment. For moderate sized sequence collections (10s of thousands) the method executes on a laptop computer within seconds or minutes, so allowing various parameters and cutoffs to be tested in repeated runs.

An artificial sequence collection was generated under a random residue substitution model in which 20 distinct equal sized families were evolved, each of which had a bias towards one amino acid type. The evolution of these sequences was constrained to maintain a minimum 33 % bias towards their starting homopolymer which is sufficient to allow alignment within a family but prevent alignment between families. Therefore, irrespective of the number of starting sequences, the final result should be just 20 sequences: one from each family. This metric was used to evaluate both various parameter choices for the current method and other methods.

On real sequences, the resulting sequence collection can be reduced to any extent but one would typically aim for between 10 and 100 sequences that can then be aligned by almost any multiple sequence alignment program. Having a small number of sequences also means that secondary analysis programs can then be applied easily, even when they are computationally demanding. Examples were provided for three families of differing type and size, from a very large globular protein family of over 350,000 members to a small RNA sequence family of just 400 sequences.

For each of these applications, visualisation of the alignment was produced using derived structure predictions, including *α* and *β* secondary structure, transmembrane segments and (for RNA) the base-paired stem-loops. For the TM-protein example, the TM-segments were used to improve the final alignment of the reduced family and for the RNA family, the alignment produced by the search program was retained through the reduction process.

## Conclusions

The MULSEL program described here bridges a gap between fast clustering methods with little or no capacity to align diverse sequences (being limited by the pairwise comparison of single sequences) and any number of slower multiple sequence alignment methods that use profile comparison but make no reduction in the number of sequences.

It would be possible to combine existing methods, such as CD-HIT [7] or MAFFT [9] and, say, T-coffee [45] or Clustal [46] to produce an equivalent functionality, however, a considerable amount of manual intervention would be required in the selection of clusters to combine for alignment and adding selection biases at the level of individual sequences would be almost impossible without re-coding.

By contrast, the current method provides a seamless transition from one to the other and furthermore, presents the resulting reduced sequences in a graphical manner that makes it clear if members have been misaligned or if there are sequences present that appear inconsistent. In this situation, the method is fast enough that it can be easily re-run after manual pruning or with a more restrictive set of parameters.

## Availability and requirements

All data and program source codes can be found in the Additional file 1 associated with this paper.

## Additional file

Additional file 1 Supplementary information. (PDF 80 kb)
