# Supplementary material for: Reduction, alignment and visualisation of large diverse sequence families
Source: BMC Bioinformatics. 2016 Aug 2;17(1):300. doi: 10.1186/s12859-016-1059-9 (PMC4971687; doi:10.1186/s12859-016-1059-9)
Supplement: Additional file 1 — Supplementary information. (PDF 80 kb) [file 12859_2016_1059_MOESM1_ESM.pdf]

A Supplementary information

A.1 Test data generation

An example of the sequence test data and a family alignment is shown in Figures 1 to 3: starting with an illustration of the substitution process used to generate the data (Figure 1) followed by an example of an alignment after selection for the T-rich family (Figure 2) and a corresponding dendrogram that records the clustering process (Figure 3). Only a part of the sequences are shown which were, on average, 74.2 residues long.

```
LTTTTT-TPD--TT--GTTITPTATLWTT-T-G-YNTRTWHTTNKYTTLT-WTTTK-TTT-TTTEIA-TTN
LTTTTT-TPD--TT--GTTITPTATLWTT-T-G-YNTRTWHTTNKYTTLT-WTTTK-TTT-TTTEIA-TTN
LTTTTT-TPD--TT--GTTITPTATLWTT-T-G-YNTRTWHTTNKYTTLT-WTTTK-TTT-TTTEIA-TTN
LTTTTT-TPD--TT--GTTITPTATLWTT-T-G-YNTRTWHTTNKYTTLT-WTTTK-TTT-TTTEIA-TTN
LTTTTT--PD--TT--GTTITPTATLWTT-T-G-YNTRTWHTTNKYTTLT-WTTTK-TTT-TTTEIA-TTN
LTTTTT--PD--TT--GTTITPTATLWTT-T-G-YNTRTWHTTNKYTTLT-WTTTK-TTT-TTTEIA-TTN
LTTTTT--PD--TT--GTTITPTATLWTT-T-G-YNTRTWHTTNKYTTLT-WTTTK-TTT-TTTEIA-TTN
LTTTTT---D--TT--GTTITPTATLWTT-T-G-YNTRTWHTTNKYTTLT-WTTTK-TTT-TTTEIA-TTN
LTTTTT---D--TT--GTTITPTATLWTT-T-G-YNTRTWHTTNKYTTLT-WTTTK-TTT-TTTEIA-TTN
LTTTTT---D--TT--GTTITPTATLWTT-T-G-YNTRTWHTTNKYTTLT-WTTTK-TTT-TTTEIA-TTN
LTTTTT---D--TT--GTTITPTATLWTT-T-G-YNTRTWHTTNKYTTLT-WTTTK-TTT-TTTEIA-TTN
LTTTTT---D--TT--GTTITPTATLWTT-T-G-YNTRTWHTTNKYTTLT-WTTTK-TTT-TTTEIA-TTN
LTTTTT---D--TT--GTTITPTATLWTT-T-G-YNTRTIHTTNKYTTLT-WTTTK-TTI-TTTEIA-TTN
LTTTTT---D--TTT-GTTITPIATLWTT-T-G-YNTRTIHTTNKYTTLT-WTTTK-TTI-TTTEIA-TTN
LTTTTT---D--TTT-GTTITPIATLWTT-T-G-YNTRTIHTTNKYTTLT-WTTTK--TI-TTTEIA-TTN
LTTTTT---D--TTT-GTTITPIATLWTT-T-G-YNTRTIHTTNKYTTLT-WTTTK--TI-TTTEIA-TTN
LTTTTT---D--TTT-GTTITPPATLWTT-T-G-YNTRTIHTTNKYTTLT-WTTTK--TI-TTTEIA-TTN
LTTTTT---D--TTT-GTTITPPATLWTT-T-G-YNTRTIHTTNKYTTLTSWTTTK--TI-TTTEIA-TTN
LTTTTT-L-D--TTT-GTTITPPATLWTT-T-G-YNTRTIHTTNKYTTLTSWTTTK--TI-TTTEIL-TTN
LTTTTT-L-D--TTT-GTTITPPATLWTT-T-G-YNTRTIHTTNKYTTLTSWTTTK--TI-TTTEIL-TTN
LTTTTT-L-D--TTT-GTTITPPATLWTT-T-G-YNTRTIHTTNKYTTLTSWTTTK--TI-TTTEIL-TTN
LTTTTT-L-D--TTT-GTTITPPATLWTT-T-G-YNTRTIHTTNKYTTLTSWTTTK--TI-TTTEIL-TTN
LTTTTT-L-D--TTT-GTTITPPATLWTT-T-G-YNTRTIHTTNKYTTLTSWTTTK--TI-TTTEIL-TTN
LTTTTT-L-D--TTT-GTTITPPMTLWTT-T-G-YNTRTIHTTNKYTTLTSWTTTK--TI-TTTEIL-TTN
LTTTTT-L-D--TTT-GTTITPPMTLWTT-T-G-YNTRTIHTTNKYTTLTSWTTTK--TI-TTTEIL-TTN
LTTTTT-L-D--TTT-GTTITPPMTLWTT-T-G-YNTRTIHTTNKYTTLTSWTTTK--TI-TTTEIL-TTN
LTTTTT-L-D--TTT-GTTITPPMTLWTT-T-G-YNTRTIHTTNKYTTLTSWTTTK--TI-TTTE-L-TTN
LTTTTT-L-D--TTT-GTTITPPMTLWTT-T-G-YNTRTIHTTNKYTTLTSWTTTK--TI-TTTE-L-TTN
LTTTTT-L-D--TTT-GTTITPPMTLWTT-T-G-YNTRTIHTTNKYTTLTSWTTTK--TI-TTTE-L-TTN
```

Figure 1: **Amino acid substitution process** for a fragment of the T-rich family. The composition is at the 1/3 T level at which point additional 'T' characters are introduced to maintain this level (eg: line 13 to 14).

```
   DTTTTTTYNDETICNDKTYFDKTYITQKTKTATLTTTKCTETTTFDTFEITLFDEDITYTHKTGVTTT
    TGLTTATNETTICNTKWYSK  YITQNYTTATLDTTKTTTNCTTVTHQIITTDVESITTHIAKTTTS
FTTETTTTVQFMTTTTGYAQTHS         MTRTQQKTTRYGMTTTRTKE        TRTTFHSRTTT
HTTTTNTTVAQMITTMMTTDTMS         TTQRLTTTTTTMQRKTKTDTYYKCDSIITTDTFYSVTTK
       FTTTLQTTFITTERTRTRLCTTTNTDLTVPFWTTTNTPTTSYQTTTFRPHTITITFMSTDTMTS
       FTTTLTTQTTTMHRTCRSTREMTNTTDLWFFWTTTTTYCPSYTTTPRTPHT TITFKPTHTTTT
        TTNHATTTNTTT TKTRNGNN NTTTMVGRRNRTAT     QWIIT   QLTHTTQ TSTATV
        TWTHAITPNTTGCTKTRNGNNTNTTKFVDTKTRTYT     TQQTTTTRQLTITTQNTSTTTM
AFITTIIFITTCRKKTNGTTNDTAVQVTTTTTIYYTKQQTLTFTRLLTINTTMTTD   TATI  TTTTTQ
FTETT             TTTSTQAYKTTKTGATYTDQTRTTTTRYTTVHTRTTHDGEKTAHIAKTMTTTT
 THPRTELDTTDMTHMDTTTTDTNSEWTATVGATTGTTADTGHTDITT DAFTTTPNTLTPVEKDCIALTT
 THTTAMLTTTMTTLTMTTTNEVFAL     GATITTMATTTETDTYTCDQFTTTPTTLTTVFEKHTLTTN
       TGTTHTTTQITTYMDTGT     IEKIIQ                TGITNTTFTKETTETTTTT
       THTTRQTTFQFTELTTLT     TNDKHTRKATTDDIQRTKFLTVTGMTTTTCEKPTTFTDTTT
      FTGTQHTTTTTTKSTDTIMDKTPPTFWFTNFAWTKQT          TTTTTTATTTLLFQTTTT
        TTLQPTFTTYKNITTTTDYRWWTTTWHNWYWTYTMWQFTGYENYNTTTTTTETDDTHWTTTNT
    TLTEETTWNTNTKKNETTYTRGNTTKTPNACMYRVTSGTHCMCHIIETTELTHTMTYTTATTFTKTT
   ATTTTETTE TNTKTTETTTTRCGNNKTPT  MARRVARTHIMTFHIIETKLAH MTTKTATTFTTKT
  TPTWATTTTHTHTETDMDTTTFTTNLTATGAGT       THETCTPIYGTFTTHTNTYPRVLCQITTT
 TLPTWATTTTHVTTAMELGTTTTTFTTTATGAGT       TTHQCTPIYTK  TATNHTKTWLCTITTA
```

Figure 2: **Multiple alignment of selected sequences** extracted from the T-rich sub-family after clustering a starting set of 10,000 sequences.

```
................p--- USER>>seqT100 =0= [+877+] T family seq 100  0.6737
..............p-b--- USER>>seqT1163 =0= [+282+] T family seq 1163  0.46
............p-b----- USER>>seqT1399 =0= [+105+] T family seq 1399  0.20
..........p-b------- USER>>seqT3455 =0= [+133+] T family seq 3455  0.63
..........|.....p--- USER>>seqT2996 =0= [+124+] T family seq 2996  0.59
........p-b---p-b--- USER>>seqT2842 =0= [+267+] T family seq 2842  0.13
........|.....b----- USER>>seqT2525 =0= [+183+] T family seq 2525  0.22
........|.......p--- USER>>seqT2159 =0= [+149+] T family seq 2159  0.11
......p-b-----p-b--- USER>>seqT2034 =0= [+187+] T family seq 2034  0.53
......|.......b----- USER>>seqT1824 =0= [+327+] T family seq 1824  0.24
......|.........p--- USER>>seqT5277 =0= [+52+] T family seq 5277  0.109
....p-b---p-----b--- USER>>seqT5151 =0= [+54+] T family seq 5151  0.258
....|.....b---p----- USER>>seqT4580 =0= [+128+] T family seq 4580  0.71
....|.........b----- USER>>seqT4239 =0= [+211+] T family seq 4239  0.37
....|...........p--- USER>>seqT5747 =0= [+112+] T family seq 5747  0.04
....|.......p---b--- USER>>seqT5668 =0= [+89+] T family seq 5668  0.709
....|...p---b-----p- USER>>seqT6162 =0= [+45+] T family seq 6162  0.811
....|...|.........b- USER>>seqT6074 =0= [+94+] T family seq 6074  0.044
....|...|.....p----- USER>>seqT5906 =0= [+31+] T family seq 5906  0.208
....b-p-b-p---b----- USER>>seqT5005 =0= [+130+] T family seq 5005  0.47
......|...b---p----- USER>>seqT3887 =0= [+140+] T family seq 3887  0.06
......|.......b----- USER>>seqT4058 =0= [+165+] T family seq 4058  0.72
......|.......p----- USER>>seqT5414 =0= [+79+] T family seq 5414  0.007
......|...p---b----- USER>>seqT4838 =0= [+126+] T family seq 4838  0.45
......b-p-b---p----- USER>>seqT5498 =0= [+92+] T family seq 5498  0.770
........|.....b----- USER>>seqT3151 =0= [+100+] T family seq 3151  0.28
........|.......p--- USER>>seqT6354 =0= [+2+] T family seq 6354  0.9518
........b---p---b--- USER>>seqT6263 =0= [+106+] T family seq 6263  0.15
............b-----p- USER>>seqT3694 =0= [+72+] T family seq 3694  0.615
..................b- USER>>seqT3628 =0= [+101+] T family seq 3628  0.65
```

Figure 3: **Cluster dendrogram of the T-rich family**. The family was extracted from 90,000 sequences of which approximately 4600 belonged to the T-rich family. These were scattered randomly throughout the collection by sorting on the (truncated) random number to the right. The dendrogram is to the left (on its side) with aligned `b`/`p` characters marking the point at which two sequences or profiles were aligned. The number "`[+N+]`" is the number of discarded sequences that are represented by the selected sequence.

```
final.seq  92680 100 150   4 0 2 0
-1 -1 9 3 10
-1 -1 9 3 10
0  0  0   20  10  20  780   0  1 -1
0  0  0   20  14  22  760   0  1 -1
0  0  0   20  16  24  740   0  1 -1
0  0  0   20  18  26  720   0  1 -1
0  0  0   20  20  28  700   0  1 -1
```

Figure 4: **Example parameter file.** At each stage of mini-alignment generation, `MULSEL` reads the current file of sequences and implements a full peptide-based resort of the sequence order followed by a number of stages of hierarchic multiple sequences alignment using profile/profile matching. In this example file, the first line specifies that up to 92,680 sequences can be read from the file `final.seq` in the length range 100–150 residues using a peptide size of 4. (The remaining numbers are not used). The next two lines specify two cycles of peptide sorting using blocks of sequences specified by the first two numbers (-1 -1 tells the program to use default divisions). The following three numbers are: the minimum score used in clustering, the number of top matches held per sequence and the bonus given to adjacent sequences pairs. The following five lines control the mini-alignment stages. The first three numbers specify a choice of matrix (0 0 0 = identity), the next four are the gap-penalty, the sequence range outside which pairs are not considered (span), the maximum allowed indel size (window) and the score cutoff (as ten times the sequence identity). The following three numbers are not relevant to the current implementation. This file will create mini-alignments by considering pairs scoring over 780 in five stages down to 700. A previous cycle may have run from 880 to 800 and a subsequent one from 680 to 600 and so on, down to the lowest final cutoff required.

A.2 Implementation

The program `MULSEL` (which is short for multiple sequence selection) was written in the C language and run on a Dell laptop with a Intel Core i7-3740QM CPU (2.70GHz processor) and 8GB of memory. The program requires an input file of sequences and an optional file of keword/penalty pairs. In addition, a series of parameter files need to be provided for each cycle of mini-alignment generation. These could all be the same but it is better to have a higher similarity cutoff in the initial stages. An example file is shown in Figure 8 and a complete set is included in the program tarball.

A.2.1 Program structure

The algorithm described here is implemented as a pre-processing option for the multiple alignment program `MULTAL`. It can be employed either as a filter to select a reduced set of representative sequences which can then be aligned by `MULTAL` or directly as a one-pass filter to `MULTAL` to sort the input sequences into a rough order that can be exploited by the hierarchic condensation (profile/profile) approach used by `MULTAL` (or any other multiple sequence alignment method). In the former selection protocol, (referred to as `MULSEL`) a series of mini-alignments are generated in a succession of stages, each employing the selection criteria described above. This would typically progress by dropping the mini-alignment score cutoff by 10% of sequence identity on each pass until the number of retained sequences reaches a manageable number. This is the strategy followed throughout for the examples provided in the Results section.

A.2.2 Address-space restriction

In an early application of the `MULTAL` program to the clustering of all known sequences (without selection) [1], computer memory limitation was an important concern. Luckily, at that time, the number of known sequences was just under 32,767 and so could be indexed by a `short int` (in the C computer language). Sequence databanks have grown since then but so too have computer resources and indexing using a normal length `int` (over 2 billion) is easily sufficient for the results of any databank search. However, `MULSEL` also uses integer values to code and decode sequence pairs and if 100K sequences are loaded, an address space for roughly 5 billion pairs is required that is just over the `unsigned int` length. For this reason, a limit was placed on the number of sequences of 92681. For sequence numbers greater than this, two or more limited presorts can be performed and the partially sorted lists combined for final clustering. This restriction has the advantage that the presorts can be performed in parallel.

Although it was stated above that a binary tree encoding does not restrict the length of the peptide, there is however a limit imposed by the size of variable used to store the encoded peptide. Currently this is an integer which can encode a peptide of 7 residues ($20^7 \leq 2147483647$) or an oligonucleotide of 15 bases. In the unlikely need to go beyond these limits, a "`long long int`"[1] could be used (with minimal changes to the code) or a different string-based structure, such as a radix tree.

[1]In the **C** language, a `long int` has the same size as the default `int` variable and using their unsigned form makes little difference

A.2.3 Keyword-penalty input

Key-words and their associated penalties used for biasing selection will often change between databases and with time. To permit flexibility, a file of keywords can be provided, so for example, if the annotation includes a species identifier, preferences can be included for, say, ”human” or to avoid ”bacterial”.

Like the ”SEED” identifier, the inclusion of the keyword ”SKIP” causes the sequence to be ignored. This is a useful feature for refining the selection after evaluation of the final reduced alignment, from which outliers or errors may have been identified.

A.2.4 Reversed sequence control

A set of reversed (doppleganger) sequences to be used as controls on the quality of the final selection and alignment (Results section 2.1.3) can be included by copying all (or part) of the sequence collection into the file of sequences to be processed but with the angle bracket symbol, ”>”, that prefixes their code changed to its complement: ”<”.

A.3 Minor refinements and features

A.3.1 Segmental sorting

For very large numbers of sequences, the initial order N^2 comparison of all sequences may prove difficult on limited computational resources. To alleviate this, the peptide pre-sorting stage (like the program itself) can be performed in a series of stages with each pass sorting only a local segment of the data. This will miss similarities that lie at either end of the list of sequences but is still very effective at reducing the number of sequences when the input order is not random (as is typical of most sequence databanks). The remaining widely separated sequence pairs can then be identified on subsequent iterations of `MULSEL` when a full N^2 comparison becomes less costly.

If the sequence data has no initial order, or even if families are clustered but related families are dispersed, then the segmental block sorting described above will miss similarities (or only find them at a later stage). For this situation, an alternative scheme was considered in which bands of sequence pairs were evaluated starting with the most distant pairs. For even computational load spreading, each band should have an equal area. For this, successive (n) band limits need to increase by a factor of $\sqrt{n}$ since for two points a and b ($b > a$) $a^2 = b^2 - a^2$ so $b^2 = 2a^2$ and similarly, for the next band marker c, $c^2 - b^2 = a^2$ so $c^2 = 3a^2$. For example; given 1000 sequences then bands between: 0,500,707,866,1000, generate four almost equal bands of 125,000 pairs. As there is little economy gained by computing all pairs, even in stages, the bands proposed above were applied only to the more widely separated sequence pairs using the

simple formula: $b = (iN/n)/\sqrt{i},\ \forall i \leq n$. For one pass, the full matrix of pairs is calculated, dropping with increasing passes (n), as N/n.

A.3.2 Delayed sequence loading

Even when encoded as a list of unique peptides, each sequence still requires a non-trivial amount of storage, however, because these peptide lists are used only in the pre-sorting stage, they do not need to be held at the same time as the full sequence alignments. For very large numbers of sequences, the peptide lists can be generated without the sequences being stored then after sorting, when the peptide lists have been freed, sequences can be re-read for alignment. With the computer memory size available currently, even on laptops, this device is unlikely to be needed often.

A.3.3 Peptide frequency weighting

It was considered whether to maintain a count of the frequency of each peptide in a protein that could then be used in matching. This may be important in clustering low-complexity sequences: for example; two poly-alanine sequences will have a score of just 1, or two triple repeat proteins will score just 3 (whatever the peptide length). However, for typical protein (and nucleic acid) sequences, the overhead required for storing the frequencies was considered unjustified given that such low complexity sequences rarely occur.

A.3.4 Reduced alphabets

As well a being able to vary the peptide length used for sorting, an option was provided to reduce size of the amino acid alphabet by defining equivalences between the most closely related types. These were 1) S=T and L=I, 2) D=E and N=Q, 3) V=I and R=K, 4) Y=F and A=G, and both substitutions were applied when their numeric prefix (1..4) was specified in the parameters. Besides reducing the length of the peptide lists to be compared pairwise between proteins, it was suspected that this 'softening' option might lead to the recognition of more distantly related pairs.

A.3.5 Low complexity filter

Knowing the peptide composition for each protein makes it easy to add a low-complexity filter. However, this was not implemented at the peptide pre-sorting stage but the number of unique peptides identified per protein at that stage was passed to the selection penalty assignment where it was used to give a slight bias towards more complex sequences being selected.

References

[1] W. R. Taylor. Hierarchical method to align large numbers of biological sequences. In R. F. Doolittle, editor, *Molecular Evolution: computer analysis of protein and nucleic acid sequences*, volume 183 of *Meth. Enzymol.*, chapter 29, pages 456–474. Academic Press, San Diego, CA, USA., 1990.
